# Injury severity and serum amyloid A correlate with plasma oxidation-reduction potential in multi-trauma patients: a retrospective analysis

**DOI:** 10.1186/1757-7241-17-57

**Published:** 2009-11-19

**Authors:** Leonard T Rael, Raphael Bar-Or, Kristin Salottolo, Charles W Mains, Denetta S Slone, Patrick J Offner, David Bar-Or

**Affiliations:** 1 Swedish Medical Center, Trauma Research, Englewood, CO, USA; 2 DMI Life Sciences, Inc, Greenwood Village, CO, USA; 3 St Anthony Central Hospital, Trauma Services, Denver, CO, USA; 4 Swedish Medical Center, Trauma Services, Englewood, CO, USA; 5 Swedish Medical Center, Emergency Department, Englewood, CO, USA; 6 Rocky Vista University, Parker, CO, USA

## Abstract

**Background:**

In critical injury, the occurrence of increased oxidative stress or a reduced antioxidant status has been observed. The purpose of this study was to correlate the degree of oxidative stress, by measuring the oxidation-reduction potential (ORP) of plasma in the critically injured, with injury severity and serum amyloid A (SAA) levels.

**Methods:**

A total of 140 subjects were included in this retrospective study comprising 3 groups: healthy volunteers (N = 21), mild to moderate trauma (ISS < 16, N = 41), and severe trauma (ISS ≥ 16, N = 78). For the trauma groups, plasma was collected on an almost daily basis during the course of hospitalization. ORP analysis was performed using a microelectrode, and ORP maxima were recorded for the trauma groups. SAA, a sensitive marker of inflammation in critical injury, was measured by liquid chromatography/mass spectrometry.

**Results:**

ORP maxima were reached on day 3 (± 0.4 SEM) and day 5 (± 0.5 SEM) for the ISS < 16 and ISS ≥ 16 groups, respectively. ORP maxima were significantly higher in the ISS < 16 (-14.5 mV ± 2.5 SEM) and ISS ≥ 16 groups (-1.1 mV ± 2.3 SEM) compared to controls (-34.2 mV ± 2.6 SEM). Also, ORP maxima were significantly different between the trauma groups. SAA was significantly elevated in the ISS ≥ 16 group on the ORP maxima day compared to controls and the ISS < 16 group.

**Conclusion:**

The results suggest the presence of an oxidative environment in the plasma of the critically injured as measured by ORP. More importantly, ORP can differentiate the degree of oxidative stress based on the severity of the trauma and degree of inflammation.

## Background

Evidence of oxidative stress is well established in the critically ill characterized by tissue ischemia-reperfusion injury and by an intense systemic inflammatory response such as during sepsis and acute respiratory distress syndrome [1]. An increase in oxidative stress is typically present in critically ill patients as a consequence of the over production of reactive oxygen species (ROS) and the exhaustion of the endogenous stores of antioxidants [2]. In critically ill patients, ROS can be produced from four different pathways: up regulation of the mitochondrial respiratory chain resulting in bursts of superoxide radical (O_2_^-•^) release, massive production of O_2_^-• ^by the NADPH oxidase enzyme of neutrophils and macrophages (a microbiocidal pathway), over production of O_2_^-• ^by the xanthine oxidase enzyme during ischemia, and release of redox active transition metals such as iron and copper [3].

The presence of various biomarkers of oxidative stress can be measured in critically ill patients using numerous biochemical assays [4]. In a rat model of traumatic brain injury (TBI), an increase in biochemical markers of oxidative and nitrosative stresses were recorded with a concomitant decrease in antioxidants such as ascorbic acid and glutathione [5]. Similar findings have been reported in other conditions such as acute lung injury and severe burn injury [6,7]. Obviously, measuring multiple biochemical parameters, such as total antioxidants, lipid peroxidation, free radical production, protein oxidation, and/or enzyme activity, is time consuming and impractical in a clinical setting. More importantly, this type of analysis may miss other contributing factors to the overall redox balance in a trauma patient.

Oxidation-reduction potential (ORP) in biological systems has been described as an integrated measure of the balance between total oxidants (i.e. oxidized thiols, superoxide radical, hydroxyl radical, hydrogen peroxide, nitric oxide, peroxynitrite, transition metal ions, etc.) and total reductants (i.e. free thiols, ascorbate, α-tocopherol, β-carotene, uric acid, etc.) [8]. Therefore, the amount of oxidative or reductive stress present in plasma after a traumatic insult can theoretically be monitored using an ORP electrode. Previously, we demonstrated that ORP values increased significantly in plasma collected from multi-trauma patients during the first few days of hospitalization suggesting the presence of an oxidative environment [9]. We also found higher plasma ORP values in severe TBI compared to mild TBI and healthy volunteers that positively correlated with protein oxidation [10].

To test the contribution of a traumatic insult to the amount of oxidative stress present in plasma, our study was comprised of severely- and mildly-injured multi-trauma patients based on their injury severity score (ISS). Both multi-trauma groups were compared, and healthy volunteers served as baseline controls. The goal of the study was to correlate injury severity with the ORP values measured in plasma during the course of hospitalization. Additionally, the acute phase reactant serum amyloid A (SAA) was measured and used for additional comparison purposes and ORP validation.

## Methods

### Patient population

This study received approval from the HCA-HealthOne Institutional Review Board according to the guidelines published by the HHS Office for Protection from Research Risk. Included in this study were patients with mild to moderate trauma (ISS < 16) and severe trauma (ISS ≥ 16). Healthy volunteers were also included in the study for comparison purposes. All patients enrolled in the study were admitted between January 2006 and December 2007 at Swedish Medical Center (Englewood, CO).

### Sample collection

For healthy volunteers and multi-trauma patients, whole blood was collected by venipuncture using a Vacutainer™ containing sodium heparin. For healthy volunteers, only one blood sample was collected per volunteer. For traumatized patients, blood was collected from a central venous line on an almost daily basis until discharge beginning with a sample collected within 24 hours of the initial injury (i.e. admission sample). Traumatized patients that did not have a blood sample drawn within 24 hours of the initial injury were excluded from the study. Whole blood was immediately centrifuged, and plasma was collected and aliquoted. Plasma samples were stored at -80°C for future use.

### ORP measurements

ORP measurements were recorded at room temperature using a micro Pt/AgCl combination MI-800/410 cm Redox Electrode (Microelectrodes, Inc., Bedford, NH) connected to an HI4222 pH/mV/Temperature bench meter (Hanna Instruments, Woonsocket, RI). Sample supernatants were thawed, and the ORP electrode was immersed in the sample. A reading was recorded in millivolts (mV) after the ORP value was stable for 5 seconds. All samples were measured at the same time in order to limit the amount of day-to-day variability in the ORP electrode. Plasma ORP was measured for all collected plasma samples for each patient.

### SAA LCMS analysis

All collected plasma samples from trauma patients and healthy volunteers were analyzed by HPLC (Waters 2795 Separations Module, Milford, MA, USA) coupled to positive electrospray ionization time of flight mass spectrometry (+ESI-TOF MS, LCT, Micromass, UK) using a method described previously [11]. 10 μL of each sample (pre-diluted 1:10 in dH_2_O) was injected onto a YMC-Pack Protein-RP HPLC column (Waters, Milford, MA, USA) heated to 50°C. A 20 minute linear gradient from 10 to 40% B using water/0.1% trifluoroacetic acid (A) and AcN/0.1% TFA (B) was utilized with a flow rate of 1 mL/min.

For serum amyloid A (SAA), the MS spectrum was deconvolved to the uncharged, parent mass using MaxEnt 1 software (Micromass, UK). The retention time of SAA was identified using a purified SAA standard (Sigma-Aldrich, USA). The parent mass spectrum was then integrated, and the areas of each species of SAA were calculated using an advanced, proprietary MS integration software package developed in-house. The areas were added to give a total SAA area.

### Statistical analysis

Patient demographics, ORP data, and SAA levels are reported as mean ± standard error of the mean (SEM). A one-way ANOVA was used to compare demographics, ORP data, and SAA levels to test for significant differences (p < 0.05) using a Tukey-Kramer adjustment for multiple comparison testing (Mathworks, Natick, MA). All graphical data was generated using Matlab R14 (Mathworks, Natick, MA).

## Results

### Patient demographics

All patients enrolled in the study were admitted between January 2006 and December 2007 at Swedish Medical Center (Englewood, CO). A total of 119 multi-trauma patients and 21 healthy volunteers comprised the study group. Two groups were included in the trauma group: 41 multi-trauma patients with an ISS < 16 and 78 multi-trauma patients with an ISS ≥ 16 (Table 1). All three groups were age and gender matched. Overall, there were more chest, head, and neck/spine injured patients in the ISS ≥ 16 group while more external injuries (i.e. lacerations, burns, abrasions, etc.) were seen in the ISS < 16 group. In the ISS ≥ 16 group, 61.5% of the patients were ventilated, and 30.8% of the patients expired. As expected, the length of stay (LOS) for the ISS ≥ 16 group (9.6 days ± 0.8) was higher compared to the ISS < 16 group (4.7 days ± 0.7, p < 0.05). For the ISS < 16 group, an average of 3 samples was collected per patient during their course of hospitalization. For the ISS ≥ 16 group, about 5 samples were collected per patient during their course of hospitalization.

**Table 1:** Patient Demographics

	*Healthy Volunteers*	*ISS < 16*	*ISS ≥ 16*
N	21	41	78
Age (years)	40.1 ± 2.1	44.2 ± 3.2	42.6 ± 2.2
Females	17	15	23
Injury Severity Score (ISS)	-	7.8 ± 0.6	29.4 ± 1.3
Length of Stay (LOS)	-	4.7 ± 0.7	9.6 ± 0.8
ICU LOS	-	0.8 ± 0.3	5.9 ± 0.7
Complications (% of patients):			
-Sepsis	-	0 (0%)	5 (6.4%)
-ARDS	-	0 (0%)	3 (3.8%)
-Pneumonia	-	1 (2.4%)	16 (20.5%)
-Other respiratory	-	0 (0%)	4 (5.1%)
-DVT	-	0 (0%)	2 (2.6%)
			
Site of injury (% of patients):			
-Neck/spine	-	4 (9.8%)	33 (42.3%)
-Abdominal/pelvic	-	8 (19.5%)	17 (21.8%)
-Chest	-	6 (14.6%)	24 (30.8%)
-External	-	24 (58.5%)	39 (50.0%)
-Limbs	-	10 (24.4%)	21 (26.9%)
-Face	-	5 (12.2%)	18 (23.1%)
-Head	-	20 (48.8%)	47 (60.3%)
			
Patients on ventilator (% of patients)	-	6 (14.6%)	48 (61.5%)
Deaths (% of patients)	N/A	0 (0%)	24 (30.8%)

Patient demographic data is reported as mean ± standard error of the mean (SEM). No statistical significance was measured between the three groups for age and number of females.

### Plasma ORP measurements

**Figure 1:**
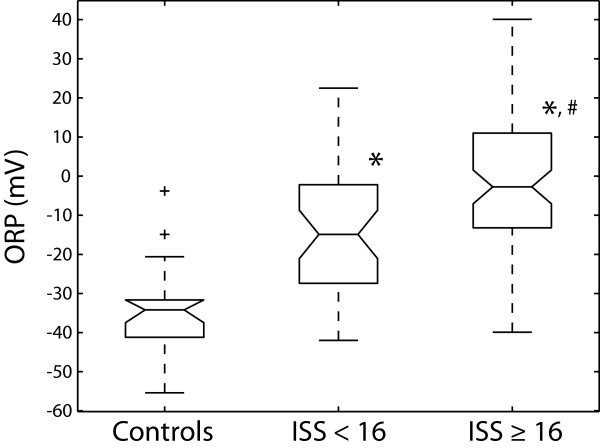
**Box plots of plasma maxima oxidation-reduction potential (ORP) measurements in healthy volunteers and multi-trauma patients**. The ORP data pertaining to healthy volunteers is labeled "Controls". The multi-trauma groups were divided into mild trauma with an injury severity score (ISS) < 16 and severe trauma with an ISS ≥ 16. The maximum ORP level was measured for both multi-trauma groups. Outliers (i.e. ± 2 standard deviations) for each group are labeled with a plus sign (+). ORP values are expressed in millivolts (mV). Statistical significance (p < 0.05) versus the control group or ISS < 16 group is indicated with an asterisk (*) or number sign (#), respectively.

Plasma ORP was measured in all collected plasma samples. An ORP maximum was assigned to the plasma sample with the highest ORP value for a particular patient during the course of hospitalization. A statistically significant difference (p < 0.05) was observed between the ISS < 16 (-14.5 mV ± 2.5) and ISS ≥ 16 multi-trauma groups (-1.1 mV ± 2.3) for the ORP maxima (Fig. 1). The ORP maxima occurred on different days for the ISS < 16 (2.9 days ± 0.4) and ISS ≥ 16 multi-trauma groups (4.7 days ± 0.5). After the ORP maxima was reached for a particular patient, ORP values for the subsequent plasma samples steadily decreased until discharge approaching the average plasma ORP of healthy volunteers (data not shown). Both multi-trauma groups had significantly higher ORP maxima values than healthy volunteers (-34.2 mV ± 2.6).

### Plasma SAA levels

**Figure 2:**
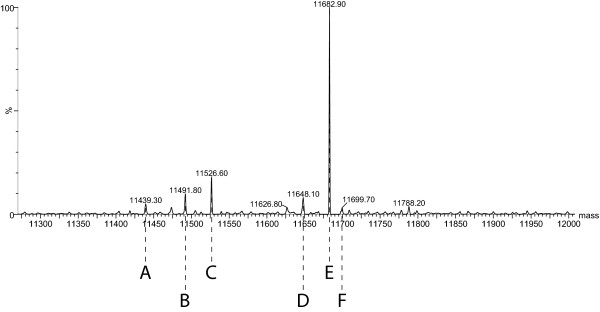
**Representative deconvolved MS spectra for serum amyloid A (SAA) in the plasma of a critically ill patient**. SAA identification: A) native SAA minus arginine-serine from the N-terminus (M^+ ^= 11,439); B) native SAA minus 35 Da and arginine from the N-terminus (M^+ ^= 11,492); C) native SAA minus arginine from the N-terminus (M^+ ^= 11,527); D) native SAA minus 35 Da (M^+ ^= 11,648); E) native SAA (M^+ ^= 11,683); and F) methionine oxidation of native SAA (M^+ ^= 11,700).

Serum amyloid A (SAA) levels were measured by LCMS analysis in conjunction with a proprietary MS spectra integration software package developed in-house. Multiple species of SAA were integrated, and total area of each species was added to give a total SAA area. As fig. 2 shows, the species included in the analysis were: SAA minus arginine-serine from the N-terminus (peak A, M^+ ^= 11,439), SAA minus arginine from the N-terminus (peak C, M^+ ^= 11,527) and minus 35 Da (peak B, M^+ ^= 11,492), native (peak E, M^+ ^= 11,683), native minus 35 Da (peak D, M^+ ^= 11,648), and methionine oxidation of native (peak F, M^+ ^= 11,700). Some of these post-translational modifications of SAA have been described previously[12]

**Figure 3:**
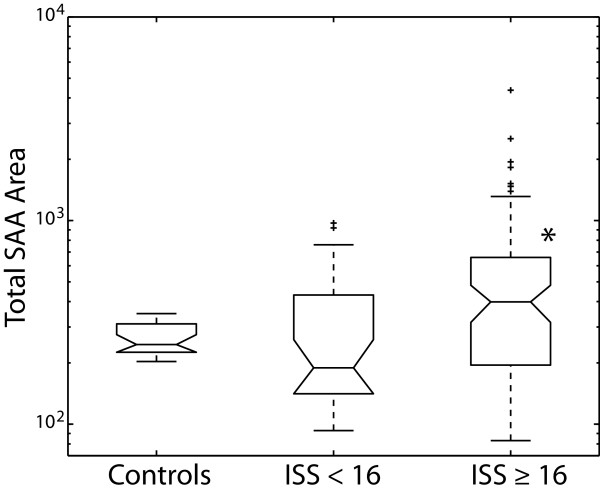
**Box plots of total serum amyloid A (SAA) levels in healthy volunteers and ORP maxima plasma samples of multi-trauma patients**. For both multi-trauma groups, total SAA levels were measured in plasma samples that recorded the maximum ORP value. Outliers (i.e. ± 2 standard deviations) for each group are labeled with a plus sign (+). Total SAA levels were calculated by adding the area under the curve (AUC) for the 6 major species of SAA (see Figure 2). Statistical significance (p < 0.05) versus the control group is indicated with an asterisk (*).

In Fig. 3, SAA data is only reported for those plasma samples that have the maxima ORP value for a particular patient. Therefore, there is only one plasma SAA value for each patient. Total SAA area was significantly greater in the ISS ≥ 16 multi-trauma group (590 ± 74) compared to the ISS < 16 multi-trauma group (310 ± 38) and healthy volunteers (265 ± 10) (Fig. 3). Interestingly, the SAA maxima occurred for the ISS < 16 and ISS ≥ 16 multi-trauma groups at 3.3 days (± 0.8) and 4.5 days (± 0.5), respectively. This is statistically similar to the ORP maxima days for the ISS < 16 (3.3 days ± 0.8) and ISS ≥ 16 multi-trauma groups at 2.9 days (± 0.4) and 4.7 days (± 0.5), respectively. Therefore, we felt justified to use the SAA levels in the ORP maxima plasma samples as an accurate measurement of SAA maxima levels. Additionally, the correlation between the ORP maxima day and SAA maxima day further validates the importance of plasma ORP maxima.

## Discussion

The occurrence of oxidative stress in critically ill patients is associated with a poor prognosis. However, no recommendation for the measurement of a single parameter of oxidative stress (i.e. lipid peroxidation, antioxidant levels, enzyme activities, etc.) can be given because the individual assays described do not allow the definition of an overall "oxidative status" for critically ill patients [13]. In the literature, it has been suggested that to obtain the best evaluation of the level of oxidative stress in a patient, a maximum of these parameters should be measured [14]. However, the measurement of even some of these parameters is time consuming and therefore impractical in a clinical setting. In previous studies, we have demonstrated the use of oxidation-reduction potential (ORP) in assessing the amount of oxidative stress in the plasma of critically ill patients and correlating with plasma paraoxonase-arylesterase activity and plasma protein oxidation [9,10]. Here, we show a positive correlation between injury severity and serum amyloid A (SAA) levels with plasma ORP in critically ill patients.

Our study suggests a positive correlation between the degree of oxidative stress in plasma as measured by our ORP electrode and severity of injury in critically ill patients. In agreement with our findings, overall plasma total antioxidant capacity has been negatively correlated with injury severity (as measured by APACHE III scores) in patients admitted to the ICU [15]. Additionally, increased plasma malondialdehyde levels are associated with poor outcome in critically ill patients with a higher level measured in non-survivors than in survivors at the time of admission [16]. In a study of severely septic patients with secondary organ dysfunction, patients who survived appeared to increase spontaneously their plasma antioxidant potential values to normal or even supranormal values during the course of hospitalization where patients who expired did not [17]. Using an HPLC method, Schorah and colleagues measured a significantly lower plasma ascorbic acid level in ICU patients compared to healthy control subjects that was associated with the severity of the illness [18]. Similarly, in head trauma and hemorrhagic stroke patients, plasma ascorbic acid levels were significantly inversely correlated with GCS scores and the major diameter of the brain lesion [19].

We also demonstrated a positive correlation between the ORP maxima and the serum amyloid A (SAA) maxima in our study. SAA is a multifunctional protein involved in cholesterol transport and metabolism, and in modulating numerous immunological responses during inflammation and the acute phase response to infection, trauma, or stress [20]. SAA concentrations in severe burn patients with complications compared to those without complications were significantly higher three days after injury [21]. This is in agreement with our measurement of an SAA and ORP maxima between 3 and 5 days in our patient pool. In a rat model of repeat mild traumatic brain injury (mTBI), Tavazzi and colleagues demonstrated that mTBI spaced 3 days apart resulted in maximal increases in oxidative and nitrosative stresses [5]. In our mild trauma group (i.e. ISS < 16), we recorded our ORP and SAA maxima around day 3 suggesting that an additional trauma on this day could result in maximal oxidative damage to an already compromised patient.

In healthy humans, antioxidants are present in excess to deal with the constant production of reactive oxygen species (ROS) within the body. Indeed, the production of ROS plays a role in the regulation of many intracellular signaling pathways that are important for normal cell growth and inflammatory responses that are essential for host defense [22]. Therefore, simply trying to scavenge ROS with antioxidant therapy is potentially harmful. Indeed, antioxidant therapy in critical illness has given mixed results with either no effect, a beneficial effect, or even a detrimental effect on clinical outcomes [3,23].

There are several reasons to explain the discrepancies observed in clinical studies regarding the prophylactic administration of antioxidants. First, increased oxidative stress can be desirable for some cell functions as mentioned before, and the importance of ROS in the regulation of these functions during critical illness is only partially understood. Second, the amount of administered antioxidants required to restore the antioxidant capacity is not accurately known and may vary according to the clinical situation. Finally, and perhaps most important, is the issue of timing of antioxidant administration. Lovat and Preiser suggest that the repletion of antioxidants would probably achieve a greater efficacy if given before a massive oxidative injury such as major surgery, shock, or severe sepsis [3]. Indeed, delayed treatment with antioxidants may not be an effective approach to their use. For example, in studies on sepsis, early administration of antioxidants resulted in a greater beneficial effect whereas, by 12 hours, the full-blown hemodynamic and metabolic effects of endotoxin infusion were well established resulting in no beneficial antioxidant effect [23,24].

The problem of timing of antioxidant administration could potentially be resolved by measuring plasma ORP. Changes in plasma ORP could give a clinician an early warning of a patient's declining condition. Additionally, if an antioxidant is administered, plasma ORP could assess the efficacy of said treatment and whether a different antioxidant should be used if no effect is observed with the first choice antioxidant. Plasma ORP monitoring could also help determine if the dose of antioxidant used is appropriate. Too much administered antioxidants can result in an equally deleterious event called "reductive stress" [25].

Our findings demonstrate the clinical value of plasma ORP monitoring in multiple ways. First, measuring plasma ORP combines all indices of oxidative stress and integrates them into a quick, clinically practical test. Second, plasma ORP correlates with injury severity and degree of inflammation. Third, plasma ORP monitoring could alert a physician of a patient's worsening condition before visual confirmation (e.g. changes to heart rate, respiration, etc.) occurs. Finally, the effect of treating oxidative stress with antioxidants or other therapeutics could be monitored in a critically patient and the dosage adjusted accordingly. Of course, the present ORP system used in this study can not be used at the bedside. Ideally, an ORP monitoring system similar to the bedside monitoring of heart rate, respiration, etc., would have to be developed to maximize the clinical benefit of measuring ORP.

## Conclusion

Our study demonstrates the presence of an oxidative environment in the plasma of critically ill patients using ORP. More importantly, we have shown a significant association between plasma ORP and injury severity. Indeed, regarding the components that comprise an antioxidant system, plasma cannot be viewed as a simple chemical, but instead a complex mixture of various components that all contribute to ORP. Therefore, the measurement of individual components is unlikely to yield a complete picture of the *in vivo *situation. We believe ORP monitoring makes it clinically possible to assess oxidative stress within a patient without the time-consuming, clinically impractical method of measuring multiple biomarkers of oxidation. A limitation of monitoring only plasma (i.e. extracellular) ORP could miss redox changes in the lipid or intracellular compartments that may be of greater importance. However, since plasma provides antioxidants to these compartments, measuring plasma ORP should give an indication of oxidative stress in a critically ill patient. Clearly, monitoring plasma ORP has the potential clinical utility in assessing the degree of oxidative stress, inflammation, severity of injury, and efficacy of antioxidant treatment in critically ill patients. More importantly, the simplicity and rapidity of measurement could make ORP monitoring a useful clinical tool.

## Abbreviations

ORP: oxidation-reduction potential; ISS: injury severity score; SAA: serum amyloid A

## Competing interests

LTR, RBO, KS, and DBO are employed by DMI Life Sciences, Inc. and have stock options through DMI Life Sciences, Inc. RBO and DBO own stock and have patent applications pending for described ORP technology. Remaining authors declare that they have no competing interests.

## Authors' contributions

LTR carried out the ORP measurements, participated in LCMS analysis of SAA, and drafted the manuscript. RBO carried out the LCMS analysis of SAA, statistical analysis, and produced the figures. KS provided the patient demographics. CWM, DSS, and PJO identified patients to enroll in the study and provided medical expertise. DBO was the primary investigator and oversaw the completion of the study.
